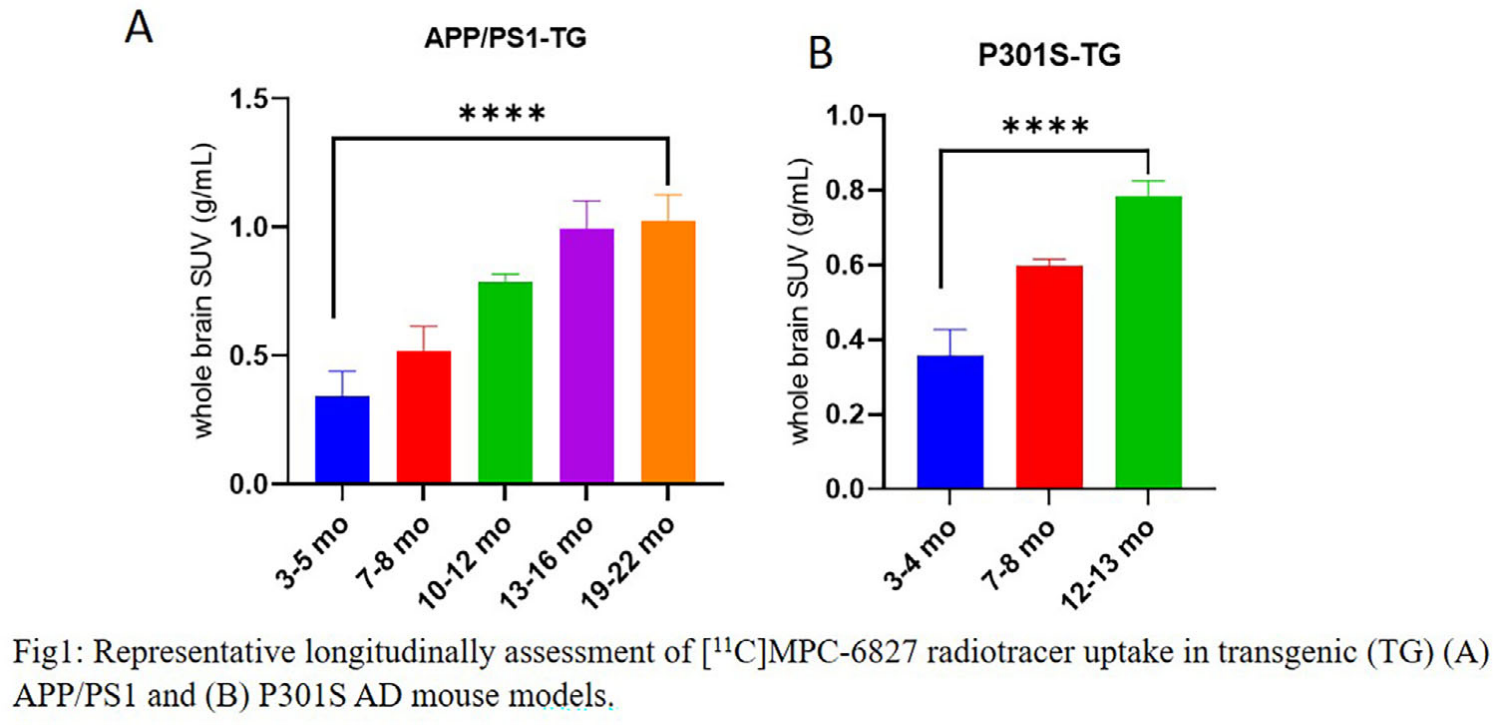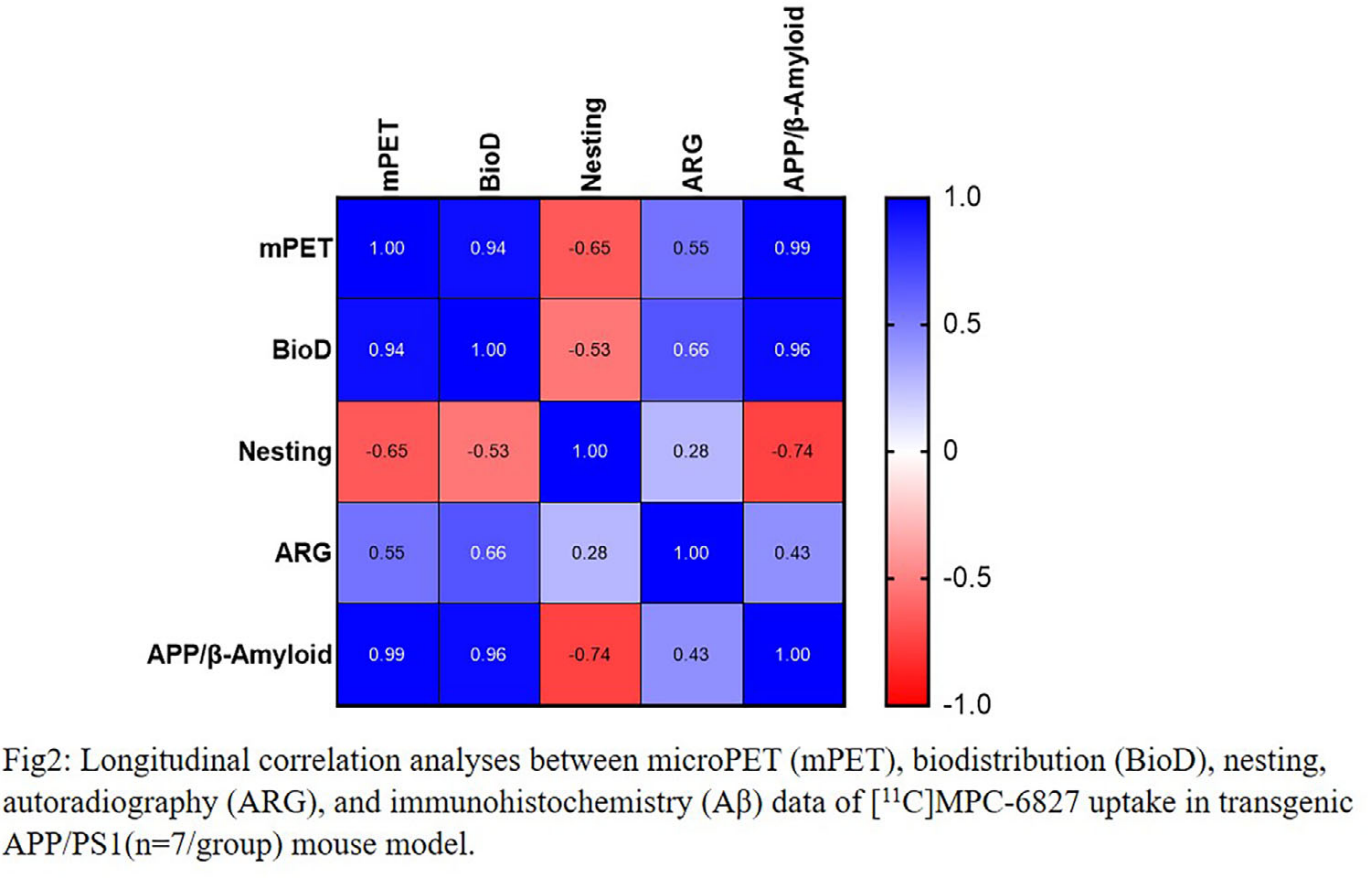# Exploring Microtubule Dynamics in Alzheimer’s Disease: Longitudinal Assessment Using [11C]MPC‐6827 PET Imaging in APP/PS1 and PS301S Mouse Models

**DOI:** 10.1002/alz.093929

**Published:** 2025-01-09

**Authors:** Naresh Damuka, Krishna Kumar Gollepalli, Ivan Krizan, Bhuvanachandra Bhoopal, Mack Miller, Ojasvi Deep, Nagaraju Bashetti, JV Shanmukha Kumar, Miranda E. Orr, Shannon L Macauley, Christopher T Whitlow, Akiva Mintz, Samuel N. Lockhart, Suzanne Craft, Kiran K. Solingapuram Sai

**Affiliations:** ^1^ Wake Forest University School of Medicine, Winston‐Salem, NC USA; ^2^ Koneru Lakshmaiah University, Guntur, AP India; ^3^ University of Kentucky, Lexington, KY USA; ^4^ Columbia University Medical Center, New York, NY USA

## Abstract

**Background:**

Brains affected by Alzheimer’s disease (AD) exhibit senile plaques containing amyloid beta (Aß) peptides and neurofibrillary tangles, formed when tau becomes hyperphosphorylated and disengages from microtubules (MTs). Early instability in MTs is observed in the AD process, emphasizing its significance in connecting the hallmark pathologies of Aß/tau‐based degenerative events. While current Aß and tau PET approaches can characterize disease lesions, they fall short in capturing earlier molecular events. MT‐PET aims to address this gap by targeting these early events, providing crucial insights into AD pathophysiology. The PET radiotracer, [11C]MPC‐6827, capable of tracking and quantifying MT‐based changes in real‐time and in vivo, addresses an urgent unmet need and facilitates the monitoring of MT changes in the early stages of AD. Here we present for the first time a comprehensive longitudinal PET imaging study of [11C]MPC‐6827 in Aß‐overexpressing (APP/PS1) and tau‐overexpressing (P301S) mouse models of AD.

**Methods:**

Longitudinal [11C]MPC‐6827 PET imaging, biodistribution studies, autoradiography, and behavioral assessments were conducted at multiple time points in APP/PS1 (3 to 22 mo old) and P301S (3 to 13‐mo‐old) transgenic (TG) and age‐matched wild‐type (WT) mice (n = 7/group). Whole‐brain standard uptake values for PET, %injected dose/gram for biodistribution, specific brain uptake for autoradiography, and nesting weight for cognitive assessments were determined and PET correlations for TG mice at different age‐points were calculated.

**Results:**

Longitudinal PET imaging demonstrated a 1.3‐fold (***p<0.0001) and 0.9‐fold (**p<0.005) higher brain uptake in APP/PS1 TG mice from 3 to 22 mo and P301S TG mice from 3 to 13 mo respectively (Fig1) and uptake increased with increasing Aß and tau loads in AD brains. Longitudinal PET data positively correlated with biodistribution (Pearson r = 0.94), and autoradiography results (r = 0.55) and negatively correlated with cognitive data (r = ‐0.65) in APP/PS1 mice (Fig2).

**Conclusion:**

Increased [11C]MPC‐6827 brain PET uptake with both increasing Aß and tau depositions longitudinally in AD brains (along with corroborating biodistribution and autoradiography results) clearly demonstrate the role of early MT dysregulations in Aß and tau‐based neurodegeneration processes. These findings highlight the potential of [11C]MPC‐6827 PET as a powerful tool for monitoring MT disintegration early‐on. Ongoing experiments are exploring translational potential in humans.